# Relationship between the body mass index and the ponderal index with physical fitness in adolescent students

**DOI:** 10.1186/s12887-022-03296-0

**Published:** 2022-04-27

**Authors:** Marco Cossio-Bolaños, Rubén Vidal- Espinoza, Camilo Urra Albornoz, José Fuentes-Lopez, Lucila Sánchez-Macedo, Cynthia Lee Andruske, José Sulla-Torres, Rossana Gómez Campos

**Affiliations:** 1 Departamento de Ciencias de La Actividad Física, Universidad Católica del Maule, Talca, Chile; 2 Universidad Católica Silva Henríquez, Santiago, Chile; 3 Escuela de Ciencias del Deporte Y Actividad Física, Facultad de Salud, Universidad Santo Tomás, Talca, Chile; 4 Instituto de Investigación en Ciencias de La Educación (IICE), Universidad Nacional del Altiplano de Puno, Puno, Perú; 5 Centro de Investigación CINEMAROS, Arequipa, Perú; 6 Universidad Católica de Santa María, Arequipa, Perú; 7 Departamento de Diversidad E Inclusividad Educativa, Universidad Católica del Maule, Talca, Chile

**Keywords:** Body mass index, Ponderal index, Adolescents, Physical aptitude, Altitude

## Abstract

**Background:**

The relationship between the Body Mass Index (BMI) with physical fitness in children and adolescent populations from diverse regions are consistent. However, the relationship between the Ponderal Index (PI) with physical fitness, based on what is known to date, has not been examined in depth. The objective was to evaluate the relationships between BMI and PI with three physical fitness tests of students living at moderate altitudes in Peru.

**Methods:**

A descriptive correlational study was carried out with 385 adolescents, between the ages of 10.0 to 15.9 years old, from the province of Arequipa, Peru. Weight, height, and three physical fitness tests (horizontal jump, agility, and abdominal muscle resistance) were evaluated. BMI and PI were calculated, and they were, then, categorized into three strata (low, normal, and excessive weight). Specific regressions were calculated for sex, using a non-lineal quadratic model for each item adjusted for BMI and PI.

**Results:**

The relationship between BMI and PI with the physical tests reflected parabolic curves that varied in both sexes. The regression values for BMI in males oscillated between R^2^ = 0.029 and 0.073 and for females between R^2^ = 0.008 and 0.091. For PI, for males, it varied from R^2^ = 0.044 to 0.82 and for females, from R^2^ = 0.011 to 0.103. No differences occurred between the three nutritional categories for BMI as well as for PI for both sexes (p range between 0.18 to 0.38), as well as for low weight (BMI vs PI), normal weight (BMI vs PI), and excessive weight (BMI vs PI) (p range between 0.35 to 0.64).

**Conclusions:**

BMI showed inferior quadratic regressions with respect to the PI. In addition, physical performance was slightly unfavorable when it was analyzed by BMI. PI could be a useful tool for analyzing and predicting physical fitness for adolescents living at a moderate altitude since it corrects for the notable differences for weight between adolescents.

## Background

For decades, throughout the world, Body Mass Index (BMI) has been considered to be a tool for detecting overweight and obesity [1, 2]. This indicator has been included as a component of physical fitness related to health in children and adolescents in diverse regions of the world [3–5].

Actually, the relationship among BMI and physical fitness indicators among youth are commonly analyzed in terms of negative influences with regard to physical fitness due to excessive weight related to height [6]. In this sense, researchers from a number of studies have proposed that a negative linear relationship exists between BMI and physical fitness [7–9]. However, recently, the presence of a quadratic relationship exists with physical fitness tests that influence the extremes of the nutritional state when categorized by BMI (underweight, overweight, and obese) [6, 10, 11], respectively.

In fact, the relationships between BMI and physical fitness in populations of children and adolescents from diverse regions of low altitude are consistent [12]. However, from what is known to date, no study has demonstrated that BMI and/or PI are effective predictors of physical fitness tests in geographic regions of moderate and high altitudes.

Thus, researchers from various studies carried out on children and adolescents living in regions of high altitude have argued that due to short stature, BMI values could certainly lead to an exaggerated increase in weight, and this could over stimulate excess weight in individuals of short height [13–15]. In addition, altitude plays a relevant role on the physical growth of adolescent children, whose heights are generally lower than international references and even with their counterparts living at sea level [16].

Therefore, BMI as an indicator of obesity would not be applicable in populations that have notable height differences [17], and, consequently, its use and application for predicting physical fitness would be questionable.

From this perspective, some researchers suggest that weight multiplied by elevated height cubed (Ponderal Index, PI) could be an indicator of satisfactory body adiposity related to BMI [18, 19]. It is important to adjust height to the cube to correct for variations, not only for stature, but also for age and weight, especially during adolescence, where biological maturation and altitude play a relevant role in body composition for children and adolescents [20].

As a result, based on the fact that current researchers have argued that quadratic relationships exist between BMI and physical fitness [6, 10, 11], the authors of this study hypothesized that physical fitness tests could reflect greater quadric relationships with PI than with BMI, The morphological component of physical fitness generally includes anthropometric indicators (e.g., BMI, waist-height index, fat percentage, among others), which are often used to analyze the physical performance of children and adolescents. In this context, the applicability of PI could be an alternative for predicting the physical fitness test of adolescents living at moderate altitudes in Peru. Thus, the objective of this study was to evaluate the quadratic relationships between BMI and PI with three physical fitness tests [horizontal jump (HJ), agility, and abdominal muscle resistance] in adolescents living at a moderate altitude in Peru.

## Methods

### Type of study and sample

A cross-sectional descriptive study was carried out with 385 adolescents (197 males and 188 females) from the province of Arequipa, Peru. The age range varied from 10 to 15.9 years old. The students were selected probabilistically (stratified by age and sex) from three public secondary schools in the urban area of Arequipa (Peru). After sample calculation it was determined that out of 1400 students (750 males and 650 females) 385 represent 27.5% [197 (14.08 males and 188 (13.42%) females] with a 95% CI.

This city is located at 2320 m above sea level. It is situated 1090 km to the south of the capital of Peru (Lima).The climate in the city of Arequipa is predominantly dry during the months of April to November. During the months of December to March, the climate is cloudy with little rain. Throughout the year, the relative humidity varies between 46% and a maximum of 70%, and temperatures range from 10° C to 25° C [21].

The school administration approved the research study. In addition, the students’ parents and/or teachers signed informed consent. Also, each student provided informed consent to participate in the study. The research was conducted in accordance with the research project (UNSA 2017–15) and the local ethics committee, respecting the Declaration of Helsinki for Human Subjects.

The students included in the study were those who were in the established age range and performed physical education classes on a daily basis. Students who did not attend the day of the evaluations (without prior notice) and those who requested medical permission were excluded from the study.

### Procedures

Anthropometric measurements and physical fitness tests were conducted at the facilities of the three schools during school hours (8:00—12:30), Monday through Friday, and the months of April through July 2017. The evaluations included: the anthropometric measurements and afterwards the physical fitness tests (HJ, abdominal muscle resistance, and agility). During the evaluations, two assistants conducted the data collection process.

Anthropometric measurements were evaluated according to the recommendations of Steward and Marfell-Jones [22]. Body weight (kg) was assessed using an electric scale (Tanita, United Kingdom) with a scale of 0 to 150 kg and with an accuracy of 100 g. Standing height was measured with a portable stadiometer (Seca Gmbh & Co. KG, Hamburg, Germany) with an accuracy of 0.1 mm.

The anthropometric variables were measured while the students were barefoot and with the least possible amount of clothing (only a light shirt and short pants). All of the anthropometric variables were assessed on two occasions by only one evaluator (the values of the test re-test varied between R = 0.88 and 0.94). BMI was calculated using the formula: BMI = weight (kg)/height^2^ (m) and the PI = weight (kg)/ height^3^ (m).

The physical fitness tests were evaluated after a previous warm-up. These included jogging, joint mobility, and agility exercises lasting for 10 – 15 min.

The *abdominal muscle resistance* (AMR) test evaluated the strength endurance of the abdominal muscles. The test was carried out on a mat. The student was evaluated for a minute (60 s) while lying on the back with hands on the neck and the knees flexed and the help of a peer. A Casio® chronometer with a precision of (1/100 s) was used, following the recommendations of California Physical Fitness Test, PFT [23]. This test was performed twice, maintaining a rest between both executions (from 15 to 20 min).

The HJ (cm) measured the explosive force of the lower extremities [24]. A 3 m metal tape measure with an accuracy of 0.1 cm was used to measure the distance. The schoolchildren performed the jump with their feet together, the student made the jump with the greatest possible impulse to go as far as possible from the original starting line. The test was performed twice times, and the jump with the greatest distance was recorded.

The *agility* test consisted of 5 m X 10rep. Two lines were drawn (separated by 5 m) based on the description of Verschuren et al. [25]. The subject needed to run at a maximum speed from one side to the other, repeating the activity 10 times without stopping (completing a total of 50 m). At each marker, the feet needed to completely cross the line. The time (sec) it took to perform the 10 repetitions was controlled. The test was performed twice, recording the best time between the two repetitions.

To guarantee the quality control of the measurements, a test and retest was used, where the three physical fitness tests were evaluated twice by a single evaluator. The values of the technical error of measurement range from 1.0 to 2.0%.

In order to classify the nutritional categories by BMI, the cut-off points from the World Health Organization were used [26]. The PI categories were determined using the proposal of Gómez-Campos et al. [20]. Both references classified the nutritional status: *p* < 10 low, p10 to p85 normal, p85 to p95 over weight, and > p95 obese (excess weight > p85).

### Statistical analysis

The normality of the data was verified by means of the Kolmogórov-Smirnov (K-S) test. Calculations for descriptive statistics (average, standard deviation, and range) were carried out to determine the characteristics of the sample. The differences between both sexes were carried out by means of the t-test for independent samples. The differences in the low category (BMI vs PI), normal (BMI vs PI), and excess weight (BMI vs PI) were compared with the t-test for related samples. The differences between the nutritional categories were determined using one way ANOVA and Tukey’s post hoc specificity test. The regressions by sex and for each specific test with BMI and PI were carried out using the quadratic model. Each physical test (PT) was considered as a dependent variable while the BMI and PI were considered as independent variables: PT = a + b* BMI + c*BMI^2^, PT = a + b*PI + c*PI^T^, where PT: physical tests, a (intersection), b (linear coefficient), and c (non-linear coefficient). In all cases, *p* < 0.05 was considered to be significant. Calculations were carried out with Excel sheets and SPSS 18.0.

## Results

The anthropometric and the physical fitness variables are illustrated in Table 1. For body weight and standing height, no differences occurred during the initial ages (in weight at ages 10, 11, and 12 years old and height at ages 10 and 11). Then, as age advanced until 15 years old, the males were heavier and taller compared to the females (p range between 0.02 to 0.03). For the BMI and PI, no differences were observed at 10 and 11 years old. However, from age 13 to 15 years old, females presented higher values (p range between 0.015 to 0.035). For the three PF tests, significant differences occurred between both sexes (p range between 0.18 to 0.38). At all ages, the males performed better physically than the females.

**Table 1:** Characteristics of the simple studied

Age (years)	N	Weight (kg)		Height (cm)		BMI (kg/m^2^)		PI (kg/m^3^)		HJ (m)		Abdominal M. R. (rep)		Agility 10 × 5 m (sec)	
		X	SD	X	SD	X	SD	X	SD	X	SD	X	SD	X	SD
Male															
10,0–10.9	20	44.8	9.1	147	7.4	20.1	3.6	13.7	2.6	1.22 ^a^	0.21	35.3 ^a^	10.1	19.40 ^a^	1.4
11.0–11.9	38	46.2	8.6	152	6.8	20	3.6	13.2	2.6	1.29 ^a^	0.26	35.1 ^a^	8.66	19.22 ^a^	1.42
12.0–12.9	42	48.5	9.4	155.9 ^a^	7.7	19.9 ^a^	3.4	12.8 ^a^	2.2	1.34 ^a^	0.28	38.9 ^a^	9.81	18.50 ^a^	1.16
13.0–139	45	55.4 ^a^	10.4	162.8 ^a^	6.7	20.9 ^a^	3.5	12.9 ^a^	2.2	1.44 ^a^	0.29	39.5 ^a^	8.97	18.78 ^a^	2.49
14.0–14.9	39	56.9 ^a^	11.6	163.4 ^a^	7.6	21.2 ^a^	3.5	13.0 ^a^	2.1	1.51 ^a^	0.27	38.5 ^a^	8.8	18.12 ^a^	1.38
15.0–15.9	33	55.8 ^a^	7.6	166.1 ^a^	5.2	20.2^a^	2.5	12.2 ^a^	1.6	1.68 ^a^	0.28	43.9 ^a^	10.2	17.72 ^a^	1.67
Females															
10.0–10.9	15	46.6	7.6	149	5	21	3.7	14.1	2.7	1.06	0.21	28.7	7.09	21.1	1.89
11.0–11.9	41	45.4	8.2	150	5.1	20.1	2.9	13.4	1.8	1.08	0.23	29	7.18	21.1	1.87
12.0–12.9	38	49.4	9.5	152	4.8	21.3	3.5	14	2.2	1.07	0.28	31.1	7.04	20.6	1.77
13.0–139	36	52.3	7.8	154	5.6	22	2.9	14.3	2	1.14	0.23	30.5	5.93	21	1.94
14.0–14.9	27	53.4	7.2	155	4.8	22.4	3.1	14.5	2.1	1.11	0.23	30.1	8.73	20.7	1.51
15.0–15.9	31	51.6	8.3	154	5.1	21.6	2.7	14	1.7	1.12	0.17	32.1	7.61	21	1.59

*X* Average, *SD* Standard Deviation, *BMI* Body Mass Index, *PI* Ponderal Index, *HJ* Horizontal Jump, *AMR* Abdominal Muscle Resistance, *rep* repetitions, *sec* seconds, ^a^: significant differences in relation to females (p range between 0.015 to 0.03)

**Fig. 1:**
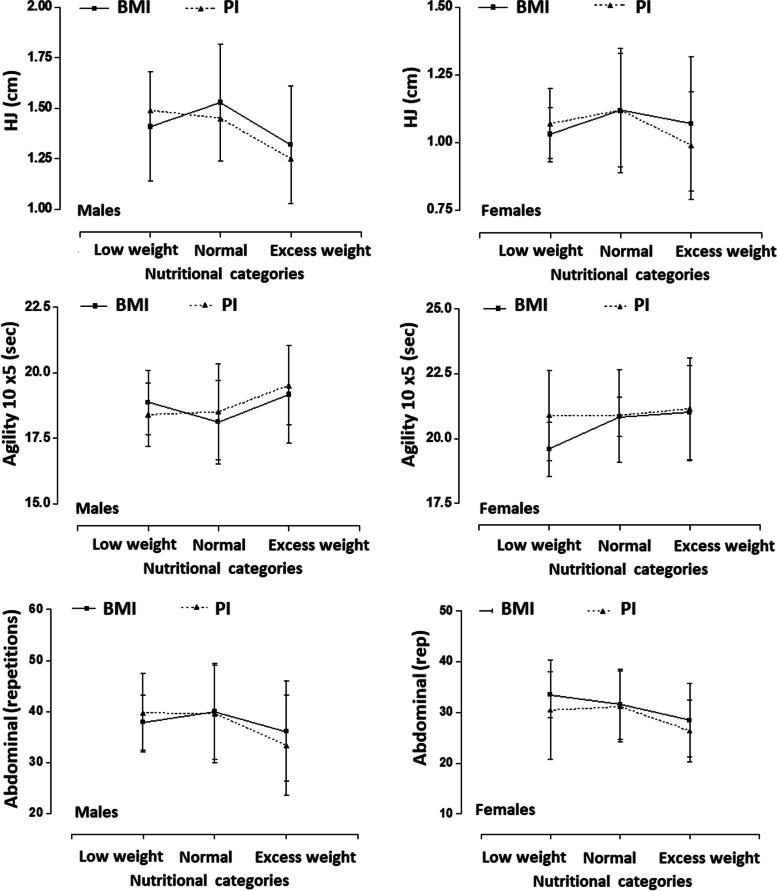
Comparison of physical fitness according to nutritional categories by BMI and PI in adolescents of both sexes

 The comparisons of the performance in the physical fitness tests based on the BMI and PI for both sexes are shown in Fig. 1. No significant differences emerged between the nutritional categories (low weight, normal, and excess weight) for BMI as well as for the PI in both sexes (p range between 0.35 to 0.64). In the same way, no significant differences were observed in the comparisons of the three physical tests in the low weight (BMI vs PI), normal (BMI vs PI), and excess weight (BMI vs PI) categories and in both sexes. In general, although no significant differences occurred, the PI showed a slightly diminished performance value, especially those classified as excess weight.

**Fig. 2:**
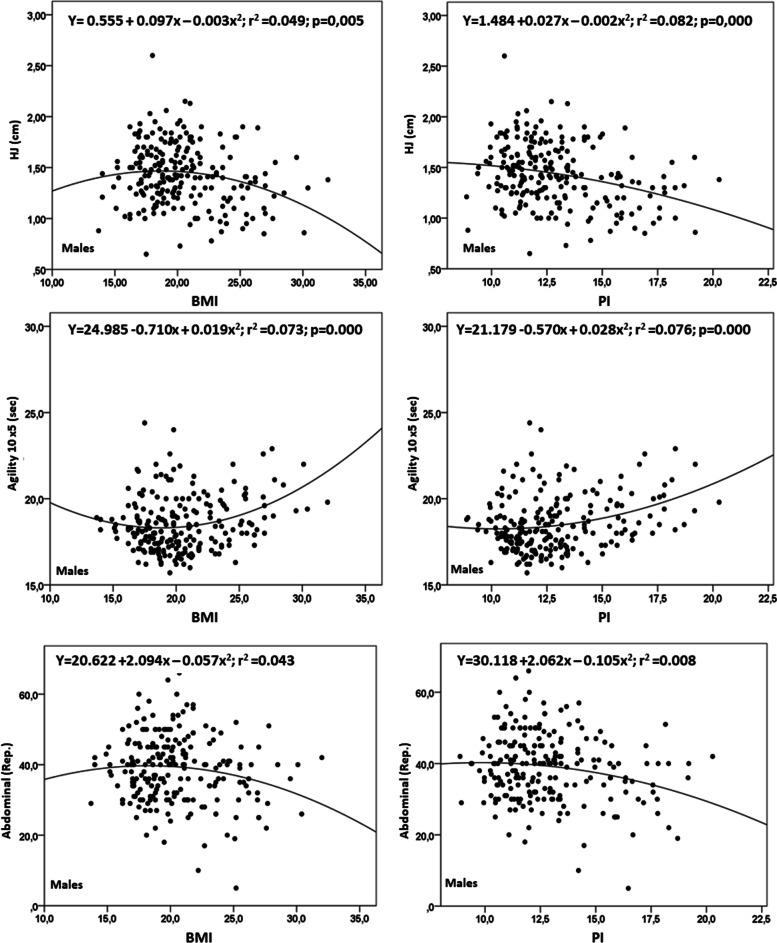
Relationship between BMI and PI with physical fitness tests of adolescent males

**Fig. 3:**
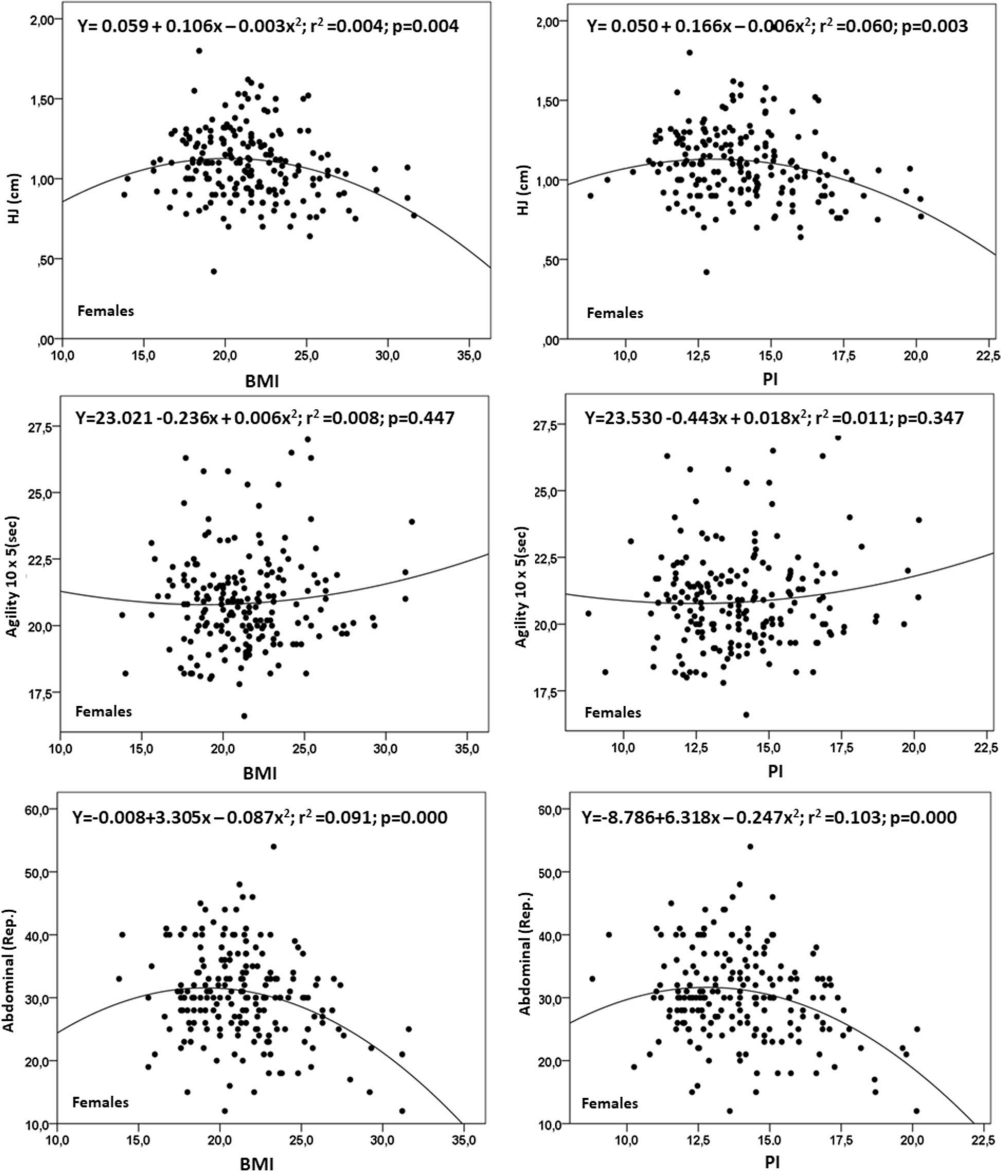
Quadratic relationship between the BMI and PI with physical fitness tests in adolescent females

Figures 2 and 3 show the correlationss and the values derived from the equations from the non-linear quadratic model for each test and sex. Quadratic relationships occurred in all of the tests. The regression values for the BMI in males oscillated from R^2^ = 0.029 to 0.073 and for females, from R^2^ = 0.008 to 0.091. For males, the PI varied from R^2^ = 0.044 to 0.082 and for females, between R^2^ = 0.011 and 0.103. In addition, physical performance benefited slightly when it was analyzed by PI in relation to BMI in all of the physical tests.

In the parabola generated by the quadratic function, maximum and minimum points for each physical test indicated the following. For males, in the HJ for BMI, it was 1.32 m, and for PI 1.57 m; and for agility for BMI, it was 18.35seg, and for PI, 18.25seg; and for abdominal muscle resistance, for BMI, it was 39.8rep, and for PI 40.3rep. For females, for the HJ for BMI, it was 0.98 m, and for PI1, 1.19 m; for agility, for BMI as well as for PI, both were 20.70seg.; and for abdominal muscle resistance, for BMI, it was 31.3rep, and for PI 31.6rep.

For the HJ, agility, and abdominal muscle resistance, the extreme values for the BMI reflected poor performance for low and elevated BMI values. However, in all of the tests, the physical performance was poorer for those who had more BMI. Despite this, when aligned with PI, poor and/or low performance was observed only with those having elevated values of PI, disappearing in those who had low PI levels. This pattern was more accentuated in males than females, especially when analyzed by PI.

## Discussion

The objective of this study was to evaluate the relationships between BMI and PI with three physical fitness tests in students living at a moderate altitude in Peru. The results from this research demonstrated that the relationships from the quadratic regression model between the anthropometric indexes (BMI and PI) varied with the physical fitness tests in both sexes. Higher correlations have also been observed in both sexes between PI and physical fitness tests in relation to BMI.

When analyzed by BMI, performance for the physical fitness tests reflected a slightly poorer performance in subjects with lower and higher levels of BMI. Additionally, the best results from the fitness tests occurred in the center of the distribution (20 kg/m^2^). In contrast, when aligned with PI, the best results from the physical fitness tests were observed in the middle and the left side of the distribution (Figs. 1, 2, and 3), specifically between 10 to 12,5 kg/m^3^. Then, performance deteriorated progressively as the PI values increased.

Furthermore, the results from the fitness tests reflected better performance when analyzed by PI rather than BMI. This indicated that the BMI underestimated the results from the fitness tests in relation to PI since height directly affects BMI [17, 20]. Body weight was not proportional to height^2^. Thus, a greater BMI was produced, underestimating the physical performance of adolescents, primarily in the HJ and slightly in agility and abdominal muscle resistance.

Consequently, based on the results obtained, the PI demonstrated itself to be a useful tool in relation to the BMI. PI was also useful for analyzing and predicting physical performance for adolescents living at moderate altitudes in Peru. In fact, authors from a number of recent studies have reported that PI is more accurate than BMI for indicating adiposity levels [2, 27], overweight, and obesity [28], and metabolic syndromes [29] in children and adolescents, independent of the geographical region researched.

Thus, PI is actually considered to be a more stable indicator than BMI, especially in children and adolescents [2]. PI has the sensitivity to distinguish the transformation of fat mass distribution in children with excess fat and the variation in fat free mass in thin children [28]. In addition, recent studies have indicated that PI adjusts better to populations in moderate and high altitudes [17, 20]. In general, BMI in these populations over estimates weight status due to short stature (product of the effect of hypoxia), increasing BMI values for each age and sex [13]. Consequently, this result prejudices even more physical performance of the adolescents studied.

Evidently, an excess of body fat has a negative influence on PF tests that require movement, propulsion of the body through space, such as races, distance jumping, lifting and sustaining the body from the floor as well as performing abdominal exercises and hanging from bars [30].

In this sense, when these negative effects are analyzed by BMI, they remained at the extremes of the distribution for the adolescents studied here. However, they disappeared when they were aligned by PI. Performance improved, specifically approximately between 10 and 12.5 kg/m^3^. While those that surpassed 12.5 kg/m^3^ tended to present poor physical performance for all of the tests.

These findings may benefit not only children and adolescents living at moderate and high altitudes, but also for those populations where notable differences exist in height [17]. This may even also include pediatric populations in the process of nutritional transition [21].

Consequently, despite the fact that BMI has been included as a component of PF in diverse regions of the world [31–33], its applicability for evaluating effects of nutritional status for populations living at altitudes has been and continues to be questionable [20]. Moreover, based on the results obtained in this research, the use of BMI as part of the morphological component of PF presents limitations when interpreting the physical performance results.

Evidently, it is necessary to conduct more research in order to confirm the results obtained in this study. Specifically, longitudinal studies are needed, those that could help verify the causal relationships. In addition, we highlight the need to increase the sample size and compare the relationships between BMI and PI with physical fitness tests for children and adolescents living in diverse geographical regions, especially controlling for confounding variables such as maturity status and physical activity levels. Furthermore, we highlight that future studies carry out allometric adjustments for the variables studied since these laws establish that shape is necessarily modified body size [17].

We would also like to emphasize that this study is one of the first that sought to verify the applicability the PI for analyzing PF for adolescents living at moderate altitude. The results obtained may be used as a base for future research and point out the possible limitations of BMI at geographical regions at altitudes.

## Conclusion

The results from this study demonstrated that BMI showed inferior quadratic regressions with regard to PI. In addition, physical performance was unfavorable when analyzed by BMI. These findings suggest that the PI may be a useful tool for analyzing and interpreting physical fitness for adolescents living at moderate altitudes since it corrects for notable differences in weight status between adolescents. In order to confirm these results, it is necessary to develop and conduct more research studies examining this topic.

## Data Availability

The generated data sets analyzed during the current study are not publicly available due to ethical approval limitations involving patient data and anonymity, but are available from the corresponding author upon reasonable request.

## Abbreviations

PI: Tri-ponderal mass index
BMI: Body Mass Index
PFT: California Physical Fitness Test
HJ: Horizontal jump
P: Significant difference
